# Controllable Fabrication of SiC@C-Fe_3_O_4_ Hybrids and Their Excellent Electromagnetic Absorption Properties

**DOI:** 10.3390/nano11123438

**Published:** 2021-12-18

**Authors:** Liqun Duan, Xiaoqing Dai, Fan Wu, Aming Xie, Jian-An Wu, Minqian Sun, Yilu Xia

**Affiliations:** 1 State Key Laboratory of Disaster Prevention & Mitigation of Explosion & Impact, Army Engineering University of PLA, Nanjing 210007, China; dxq38@sohu.com (X.D.); uniquewujianan@163.com (J.-A.W.); emmassun@hotmail.com (M.S.); xiayilu_lugongda@163.com (Y.X.); 2 School of Mechanical Engineering, Nanjing University of Science & Technology, Nanjing 210094, China; xieaming@njust.edu.cn

**Keywords:** SiC nanowires, carbon shell, Fe_3_O_4_, dielectric, magnetic, electromagnetic absorption

## Abstract

In this work, a batch of novel ternary hybrids (SiC@C-Fe_3_O_4_), characterized by SiC nanowires core, carbon shell, and adhered Fe_3_O_4_ nanoparticles were controllably synthesized via surface carbonization of SiC_nw_ followed by hydrothermal reaction. Carbon, which was derived from SiC with nanometer thickness, possesses an amorphous structure, while Fe_3_O_4_ nanoparticles are in a crystalline state. Simultaneously, the inducement of Fe_3_O_4_ nanoparticles can provide significant magnetic loss, which is well-tuned by changing the molar content of iron precursors (FeCl_3_·6H_2_O and FeCl_2_·4H_2_O). SiC@C-Fe_3_O_4_ hybrids show great electromagnetic absorption performance owing to the synergy effect of dielectric and magnetic losses. The minimum refection loss can reach to −63.71 dB at 11.20 GHz with a thickness of 3.10 mm, while the broad effective absorption bandwidth (EAB) can reach to 7.48 GHz in range of 10.52–18.00 GHz with a thickness of 2.63 mm. Moreover, the EAB can also cover the whole X band and Ku band. The outstanding performance of the obtained material implys that it is a promising candidate as an electromagnetic absorber.

## 1. Introduction

Electromagnetic (EM) waves have played an increasingly important role in many fields, which provide plenty of convenience to our daily lives. However, it is also identified that EM waves can potentially threaten human health in different forms. To address this issue, many efforts were made toward exploring EM absorption materials, which are supposed to possess multiple features, including thin thickness, lightweight, wide bandwidth, strong absorption, and good environmental adaptability [1,2,3]. Undoubtedly, it is still a huge challenge for researchers to achieve this aim unless a suitable component and appropriate microstructure are designed for EM absorption materials.

Among various EM absorption materials, silicon carbide (SiC) owns unique and impressive properties (especially for its strong workability in harsh environment conditions) and has attracted much attention in recent decades [4,5,6,7,8]. However, ordinary SiC is not suitable for EM absorption due to its poor dielectric behavior, which can be effectively improved by changing its morphology in the nanoscale [9,10,11,12] or blending with relatively higher dielectric materials (e.g., CNTs [13], graphene [14], PPy [15]). The doping or decoration with heterogeneous metal (e.g., Fe [16], Ni [17], Co [18], Al [19], Cu [20]) or compound (Fe_3_O_4_ [21], NiO [22], ZnO [23], HfC [24]) is another effective method for obtaining SiC-based absorbers. Generally, SiC-based EA materials with multiple features are designed and supposed to exhibit a more impressive performance, and consists of complicated microstructure and components at the same time. Liang et al. fabricated one dimensional SiC-Fe_3_O_4_ nano hybrids by adopting a convenient polyol technique [21]. Fe_3_O_4_ nanoparticles were used to modify SiC nanowires (SiC_nw_) in situ, which led to a significant improvement for EM absorption. The reported minimum reflection loss (RL_min_) was 51 dB at 8.6 GHz. Wu et al. synthesized one-dimensional SiC_nw_ decorated with ZnO nanoparticles and obtained a broad EAB of 6.60 GHz in range of 11.08 GH_Z_ to 17.68 GHz [23]. Except for the predominant binary hybrids, some SiC-based ternary hybrids were also investigated. Guo et al. fabricated carbon-coated Co-SiC nanomaterials (Carbon-Co-SiC) through pyrolyzing methane on nanostructured Co_3_O_4_-SiC hybrids, whose RL values below 10 dB nearly covered the whole X or Ku band [25]. To enhance the EM absorption performance of SiC_nw_, Zhou and his group [26] obtained SiC@SiO_2_@Fe_3_O_4_ hybrids using carbothermal reduction and a convenient polyol technique, in which silanol-groups-decorated SiO_2_ nanoshells (approximately 2 nm in thickness) played an important role in the growing of Fe_3_O_4_ nanoparticles by changing the weak hydrophilicity of SiC. However, their performances as ideal EA absorbers were still far from satisfying.

In this paper, a batch of novel ternary hybrids, characterized by SiC_nw_ core, carbon-shell, and adhered Fe_3_O_4_ nanoparticles (SiC@C-Fe_3_O_4_), were fabricated via surface carbonization of SiC_nw_ followed by hydrothermal reaction. The inducement of Fe_3_O_4_ nanoparticles can provide significant magnetic loss, which is well-tuned by changing the molar content of iron precursors (FeCl_3_·6H_2_O and FeCl_2_·4H_2_O), leading to an excellent EM absorption performance during the microwave band of 2–18 GHz. In addition, the mechanism of this enhancement is discussed.

## 2. Experimental Section

### 2.1. Pristine Materials and Fabrication of SiC@C Nanowires

The pristine SiC_nw_ materials (Diameter: 100~600 nm; Length: >100 μm; Density: 3.21 g/cm^3^; Purity: ~98%) were purchased from XF Nano Materials Tech Co., Ltd. (Nanjing, China), whose chemical composition detected by X-ray spectrometer (EDS) technique is shown in Table S1. For the carbon-coated SiC nanowires (SiC@C), the specific synthesis process, detailed characteristics and EM absorption performances were described in our previous work [27]. However, it is noted that the furnace body condition is fixed to the temperature of 800 °C for 1h during surface carbonization of SiC_nw_.

### 2.2. Fabrication of SiC@C-Fe_3_O_4_ Hybrids

An amount of 40mg SiC@C nanowires was added into 30 mL distilled water in an ultrasonic bath, followed by magnetic stirring to achieve a uniform suspension. Two iron precursors (FeCl_3_·6H_2_O, FeCl_2_·4H_2_O) with different molar contents (4 mmol/4 mmol, 2 mmol/2 mmol, 1 mmol/1 mmol), dissolved in distilled water, were added to the suspension with constant stirring, respectively. Then, we added NH_3_·H_2_O drop by drop, which aimed to obtain an alkaline environment of pH 9~10. Finally, the mixtures were transferred to a polytetrafluoroethylene hydrothermal reactor. The hydrothermal reaction condition in stove box was controlled at 180 °C with a dwelling time of 12 h. At last, the final products were obtained after filtering, washed with distilled water, and dried at 50 °C under vacuum. For convenience, we labeled these products as SCF4-4, SCF2-2, and SCF1-1, respectively, in which the number ratio of 4-4 indicates the 4 mmol/4 mmol molar content of FeCl_3_·6H_2_O and FeCl_2_·4H_2_O.

### 2.3. Characterization and Measurement

Morphology for SCF materials was characterized through scanning electron microscope (SEM) and transmission electron microscopy (TEM) technique. TEM tests are operated with a FE-HRTEM, Tecnai G^2^ F20UTwin, FEI microscope (FEI, Hillsboro, OR, USA) at 200 kV. SEM was performed on samples with no sputter coating and operated at 15 kV, and the energy dispersive X-ray spectrometer (EDS) was operated with an accelerating voltage of 20.0 kV. Crystalline analysis was managed by X-ray diffraction (XRD) and Raman technique, which were operated on Bruker D8 advance (Bruker, Billerica, MA, USA) and a Renishaw microspectrometer (532 nm, Renishaw, Wotton-under-Edge, UK), respectively. The relative complex permittivity (*ε*_r_) and permeability (*μ*_r_) in 2–18 GHz were obtained through a vector network analyzer (N5242A PNA-X, Agilent, Santa Clara, CA, USA). The uniform mixture of SCF samples/wax was pressed into toroidal shaped compact (Diameter: D_outer_ = 7.00 mm, D_inner_ = 3.04 mm).

## 3. Results and Discussion

**Figure 1:**
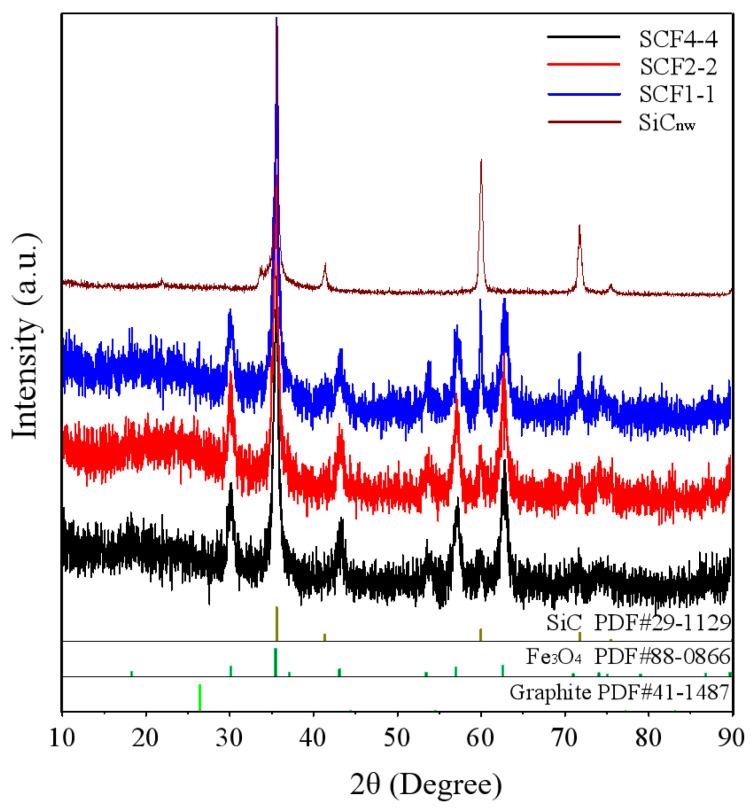
XRD spectra for all samples investigated.

For all SCF samples, several sharp diffraction peaks (35.6°, 41.4°, 60°, 71.8°, and 75.5°) can be detected, which point to the lattice plane (111), (200), (220), (311) and (222) of β-SiC (Figure 1). Different from pristine SiC, there are several new peaks (30.1°, 35.5°, 43.1°, 53.5°, 57.0°, and 62.6°) for SCF samples. These peaks correspond to the lattice plane (220), (311), (400), (422), (511), and (440) of the magnetite Fe_3_O_4_ phase (Cubic structure, PDF#88-0866), respectively. It is easy to find that the relative intensity of Fe_3_O_4_ becomes more distinct with the increasing molar content of iron precursors (FeCl_3_·6H_2_O and FeCl_2_·4H_2_O). Moreover, a broad peak at 26.5°, corresponding to the (002) reflection of the carbon phase can also be detected for SCF samples, which is inferred as an amorphous carbon phase based on the previous work [27]. A similar phenomenon also occurs for some other carbon materials with solid crystalline characteristics or an amorphous solid state [28].

**Figure 2:**
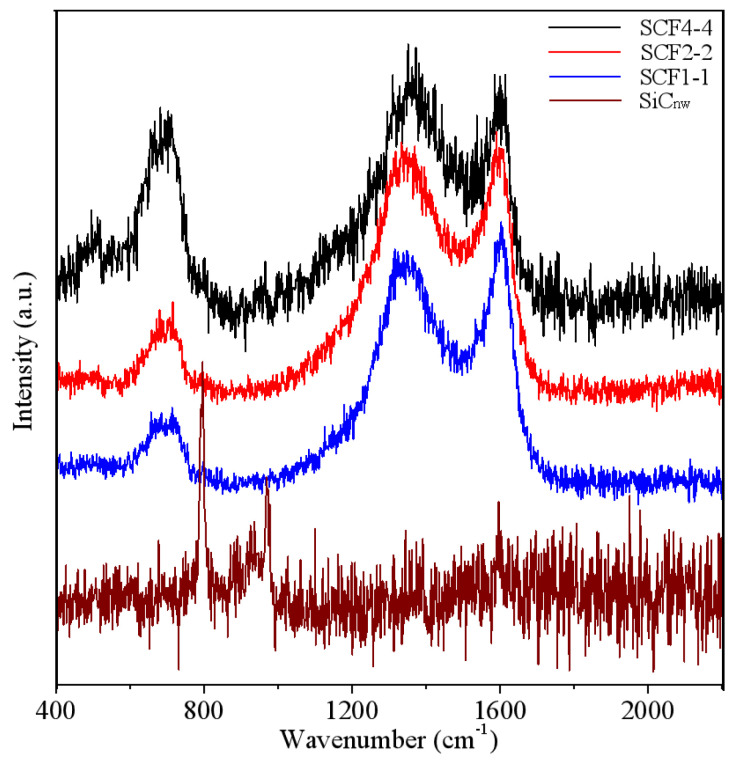
Raman spectra for all samples investigated.

Figure 2 shows that there are several Raman peaks for SCF samples, located at 680 cm^−1^, 796 cm^−1^, 973 cm^−1^, ~1342 cm^−1^, and ~1600 cm^−1^. Therein, the peak at 680 cm^−1^ points to the Fe_3_O_4_ phase, whose intensity gradually increases as a function of molar content of iron precursor, further confirming that SiC@C-Fe_3_O_4_ hybrids are successfully synthesized. Moreover, the 796 cm^−1^ peak should be attributed to the signals of transversal of SiC, while the 973 cm^−1^ peak to longitudinal optical phonons while tested. These two peaks for SCF samples are very weak or hard to detect owing to the special structures and complex compositions, which formed after all synthesis procedures. Simultaneously, the peaks at ~1342, ~1600 cm^−1^ for all SCF samples are apparent and not shown in the pristine SiC_nw_ sample. Based on our previous work, it is demonstrated that they are caused by the disordered D-mode and ordered G-mode of carbon, respectively [29,30,31]. This means the core–shell structure may be reserved well after the growing process (Figure S1) of metal oxide, which can be proven by microstructure observation.

**Figure 3:**
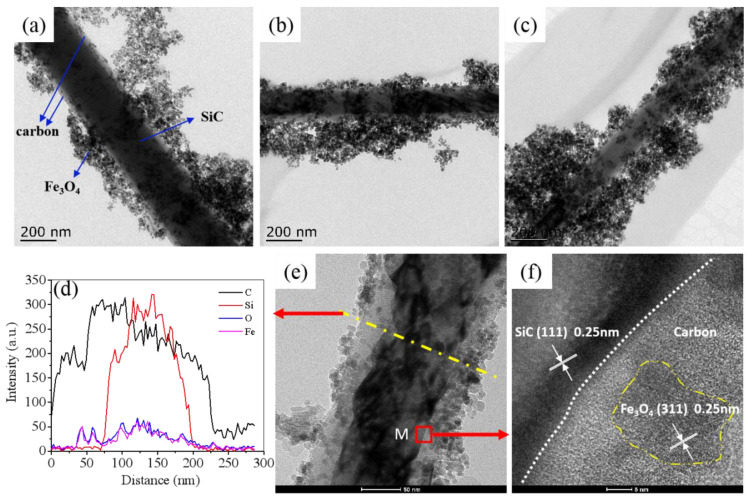
TEM images of all samples investigated: (**a**) SCF1-1; (**b**) SCF2-2; (**c**) SCF4-4; Line scanning profiles (**d**) of C, Si, O, and Fe recorded along the line labeled in HRTEM image (**e**) of SCF1-1; HRTEM image (**f**) derived from the area M in (**e**).

Figure 3 depicts TEM or HRTEM images taken from the final SiC@C-Fe_3_O_4_ samples investigated. It can be seen that SCF samples are all composed of three parts, corresponding to SiC, carbon, and Fe_3_O_4_ nanoparticles, respectively. Clearly, the hybrids are characterized by SiC core and a carbon shell, as well as the Fe_3_O_4_ nanoparticles outside the carbon shell, which is in line with results of the SEM images (Figure S2). The increase in the molar content of iron precursor led to an increase in the loading density of Fe_3_O_4_ nanoparticles on carbon (Figure 3a–c). The nanowires are composed by four elements (C, Si, O, Fe) from the line scanning profiles, as shown in Figure 3d, which is in line with the results by EDS technique (Table S1). Moreover, the C line is broader compared with the Si element, confirming the core–shell structure of SiC@C. Simultaneously, the Fe and O lines cover nearly all ranges with weak fluctuations, inferring that a large quantity of Fe_3_O_4_ nanoparticles attach to the whole surface of SiC@C. Figure 3f shows the interface between SiC core and the carbon middle shell [27], and the interface between the carbon shell and Fe_3_O_4_ nanoparticles. It is apparent that β-SiC and the Fe_3_O_4_ phase possess crystal structures, featured with plane (111) and plane (311), respectively. The predominately amorphous carbon phase has a very important effect on the nucleation of Fe_3_O_4_ nanoparticles, possibly owing to some groups (C–OH, COOH) on SiC@C that are helpful for the formation of the hydrogen band between carbon atoms and Fe_3_O_4_ [32]. Differently, Liang et al. reported that the transitional silica layer between the SiC phase and Fe_3_O_4_, was decorated by Si–OH groups, which helped form Fe_3_O_4_ nanoparticles [21]. Moreover, it is observed that the Fe_3_O_4_ nanoparticles with a relatively homogeneous size of about 11 nm cluster in some degree and seem to be loose areas, which are possibly caused by the magnetic dipole−dipole attraction [32]. Additionally, this phenomenon is more clear as the molar content of iron precursor increases, which might lead to a decline in the synergy effect on attenuating EM waves.

**Figure 4:**
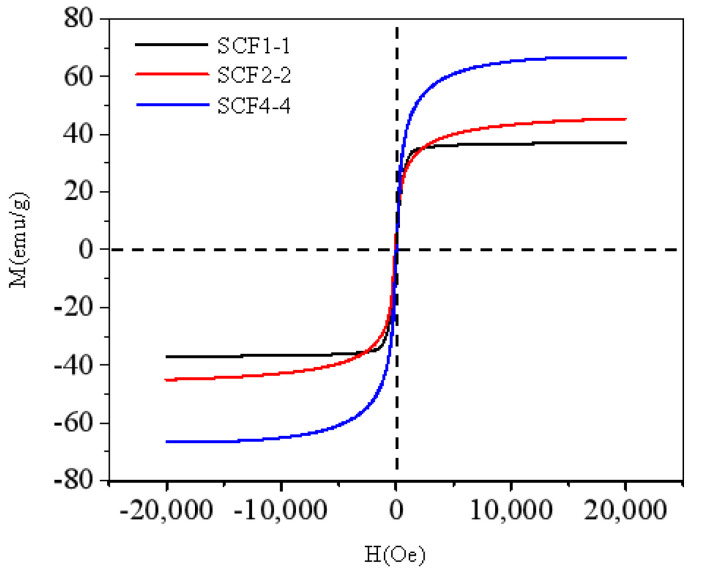
Room temperature field-dependent magnetization curves of SiC@C-Fe_3_O_4_ hybrids.

To further study the magnetic character of the SCF samples, the field-dependent magnetization curves of SiC@C-Fe_3_O_4_ samples were analyzed and drawn in Figure 4. The hybrids for SCF1-1, SCF2-2, SCF4-4 had magnetic saturation values of 36.9, 45.2, and 58.3 emu·g^−1^, respectively. Clearly, increasing the molar content of iron precursor accelerates the formation of Fe_3_O_4_ nanoparticles on the surface of the carbon shell and helps to obtain a high level of magnetic saturation. Simultaneously, it can be inferred that the strategy used in this paper is feasible for adjusting the magnetic property by changing the molar content of iron precursors.

**Figure 5:**
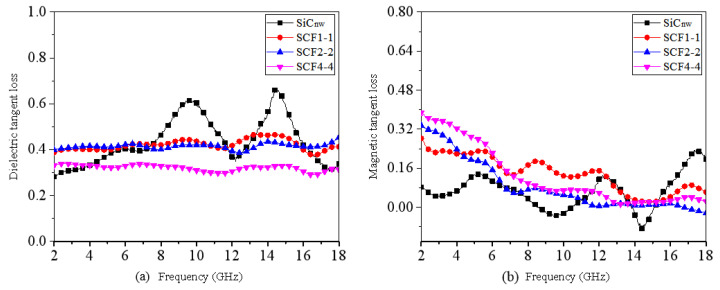
Dielectric (**a**) and magnetic (**b**) tangent loss values of SiC_nw_/wax and SCF/wax over 2–18 GHz with the same loading of 40 wt%.

The complex permittivity ($\varepsilon_{r}=\varepsilon^{\prime }-j\varepsilon^{\prime \prime }$), permeability ($\mu_{r}=\mu^{\prime }-j\mu^{\prime \prime }$) characterizations, as well as the corresponding dielectric tangent, magnetic tangent loss of different materials are shown in Figure S3. It is apparent that these materials show a similar frequency dependency. Nevertheless, both $\varepsilon^{\prime }$ and $\varepsilon^{\prime \prime }$ of SCF samples have lower values compared with SiC_nw_ in the same condition, which should be explained by nature distinction of SiC and Fe_3_O_4_. SiC belongs to typical dielectric loss material for microwaves, while Fe_3_O_4_ belongs to a magnetic material. In our previous work [27], it was found that the dielectric property of SiC_nw_ was enhanced by surface carbonization, which led to the achievement of SiC@C. It is also inferred that the controllable inducement of magnetic Fe_3_O_4_ nanoparticles may further change the dielectric properties [32,33]. It can be observed from Figure 5 that the magnetic tangent loss has a great increment for the SiC@C-Fe_3_O_4_ hybrids in comparison with SiC_nw_, while the value of dielectric tangent loss for SiC_nw_ and SCF samples stays at a similar level. However, the curves for the latter are more even than SiC. Moreover, it seems that increase the molar content of iron precursor cannot help to increase the dielectric tangent loss or magnetic tangent loss, inferring that a moderate molar proportion of iron precursor is needed to synthesize SiC@C-Fe_3_O_4_ with good dielectric and magnetic properties for microwave absorption at the same time.

**Figure 6:**
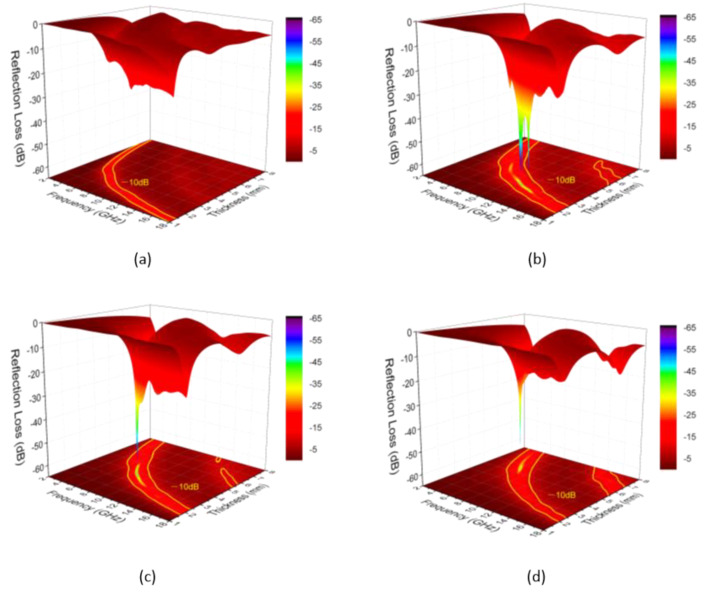
3D-RL plots of the composites over 2–18 GHz at different thickness of 1–8 mm with the same loading of 40 wt.% ((**a**): SiC_nw_; (**b**): SCF1-1; (**c**): SCF2-2; (**d**): SCF4-4).

Figure 6 shows the reflection loss of the composites/wax versus frequency. Therein, the microwave absorption performance is simulated based on the transmission line theory. Undoubtedly, SCF1-1 has a higher level of EM absorption compared with other samples. The value of RL_min_ for SCF1-1 reaches −63.71 dB at 11.20 GHz at a thickness of 3.10 mm. Its effective absorption bandwidth (EAB) less than −10 dB can reach to 6.88 GHz (11.00–17.88 GHz) with a thickness of 2.62 mm. Furthermore, the EAB of this sample can cover the X band and Ku band with thicknesses of 2.43 and 3.44 mm, respectively. However, the broadest EAB of 7.48 GHz (10.52–18.00 GHz) is achieved under a thickness of 2.63 mm when the loading of SCF1-1 decreases from 40 wt% to 30 wt% (Figure S4b). This phenomenon infers that the EM absorbing property could be further controlled by adjusting the loading of the absorbers. The value of RL_min_ for SCF2-2 and SCF4-4 can reach to −63.68 dB with a thickness of 3.86 mm when the loading is 40 wt%, and −61.12 dB with a thickness of 3.48 mm when the loading is 50wt% (Figure S4e), respectively. In conclusion, the value of RL_min_ for SiC@C-Fe_3_O_4_ is lower than SiC_nw_ and SiC@C samples. Moreover, the EAB value (7.48 GHz) for SCF1-1 at a loading of 30 wt% is also broader. Compared with other microwave attenuation materials, such as SiC@Fe_3_O_4_ [21], 3D Fe_3_O_4_-MWCNTs [33], the present materials exhibit a better behavior (lower RL*_min_* and broader EAB), demonstrating a successful strategy for microwave absorbers, as this paper details.

**Figure 7:**
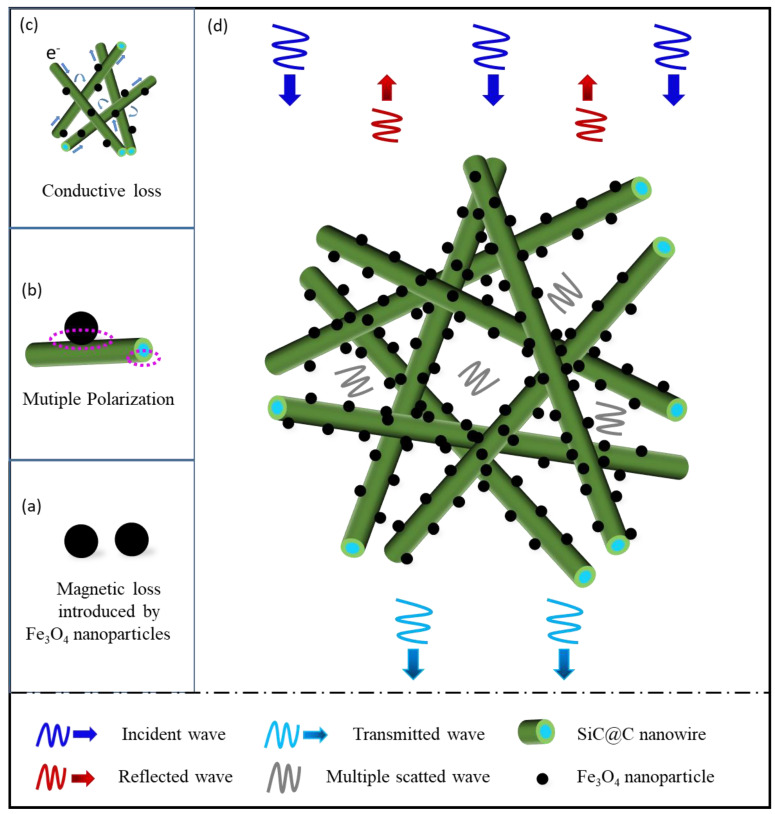
Schematic diagram of EM absorption mechanism of SiC@C-Fe_3_O_4_ hybrids ((**a**) Magnetic loss introduced by Fe_3_O_4_ nanoparticles; (**b**) Multiple polarization between carbon shell and Fe_3_O_4_ nanoparticles or SiC; (**c**) Conductive loss contributed almost by carbon; (**d**) Schematic diagram of energy dissipation among hybrids).

The possible enhancement mechanisms of EM absorption for the obtained final SiC@C-Fe_3_O_4_ hybrids are shown in Figure 7. In general, the synergy effect of dielectric and magnetic loss is a very important characteristic for excellent EM absorbers. When the input impedance of the EM absorbers reaches the impedance of free space, there is little reflection on the surface of the materials for the incident EM wave, which leads a strong attenuation of EM wave energy inside the materials, and a higher efficiency EM absorption. From this point, it is an efficient way to introduce magnetic loss to typical dielectric absorbers (Figure 7a). Without a doubt, a moderate proportion of Fe_3_O_4_ in the final SiC@C-Fe_3_O_4_ materials and rational loading in SiC@C-Fe_3_O_4_/wax is needed to get close to the ideal balance of permeability and permittivity, as well as the impedance match of the materials. Generally, the magnetic loss can originate from exchange resonance, natural ferromagnetic resonance and the eddy current effect, etc. The scale of Fe_3_O_4_ nanoparticles in this study is approximately 11 nm by measure (close to the exchange length), indicating the possible existence of exchange resonance. Figure S5 shows the frequency dependence of the C_0_ ($C_{0}=\mu^{\prime \prime }{\left(\mu^{\prime }\right)}^{-2}f^{-1}$ ) of different SCF samples with 40% loading weights as well as the SCF1-1 sample with different loading weights, whose value is approximately constant when the frequency exceeds 15 GHz, which is likely contributed to by the eddy current effect [33].

Moreover, the present SiC@C-Fe_3_O_4_ hybrids have many other features which are also beneficial for attenuating EM waves. For example, the Fe_3_O_4_ nanoparticles do not stick to the SiC_nw_ directly. There is a middle layer, namely the carbon shell on the surface of SiC_nw_, which has a relatively better conductivity and helps to increase conductivity loss (Figure 7c) [34]. Moreover, the carbon shell between the SiC and Fe_3_O_4_ phase possesses many pores (mesopores or macropores) demonstrated by previous work [27], implying that there are many defects that can cause the dipole polarization and Debye relaxations by breaking the balance of the charge distribution [35,36]. In addition, phase boundaries among the SiC_nw_ core, the porous carbon shell, and the Fe_3_O_4_ nanoparticles on the surface of carbon, possibly cause the surface charge redistribution and generate multiple interfacial polarization (Figure 7b). Furthermore, owing to the macro-porous characteristic of the SiC@C-Fe_3_O_4_ hybrids derived from three-dimensional interlaced stacking in wax, more multiple reflections and scattering will be generated to attenuate or dissipate the EM wave energy (Figure 7d). Thus, the SiC@C-Fe_3_O_4_ hybrids with core–shell microstructures can be regarded as an ideal EM absorber.

## 4. Conclusions

In conclusion, a novel and simple strategy for the controllable fabrication of SiC@C-Fe_3_O_4_ hybrids via surface carbonization and hydrothermal reaction was raised. The microstructure, permeability, as well as permittivity of the final hybrids can be well adjusted by changing the molar content of the iron precursors (FeCl_3_·6H_2_O and FeCl_2_·4H_2_O). The nanoporous carbon shell should have an important effect on the nucleation of Fe_3_O_4_ nanoparticles (with a small size of about 11 nm), possibly derived from the hydrogen band effect between the C atoms and the Fe_3_O_4_ phase. This will also help to improve the homogeneity or quality of hybrids. However, the Fe_3_O_4_ nanoparticles cluster can also be observed to some degree. This phenomenon is possibly caused by the magnetic dipole−dipole attraction, and easily occurs at a high molar content of the iron precursor. Thus, the moderate condition during synthesis is vital. Comparatively, SCF1-1 exhibits the best EM absorption performance among the final hybrids; its RL_min_ can reach −63.71 dB and EAB can reach 7.48 GHz in the range of 10.52–18.00 GHz. The excellent EM absorption performance of the final hybrids can be attributed to their good synergy of dielectric loss (including conductive loss and polarization relaxations) and magnetic loss (introduced by magnetic Fe_3_O_4_ nanoparticles), indicating a promising nanomaterial as an EM absorber.

## Data Availability

All data generated or analyzed during this study are included in this published article. Moreover, the datasets used and/or analyzed during the current study are available from the corresponding author on reasonable request.

## Supplementary Materials

The following are available online at https://www.mdpi.com/article/10.3390/nano11123438/s1, Figure S1: Schematic illustration for fabrication of SCF hybrids, Figure S2: SEM images of SiC_nw_ and SCF1-1 sample, Figure S3: The complex permittivity and permeability characterizations of SiC_nw_ and SCF samples with the same loading of 40 wt.%, Figure S4: 3D-RL plots of the SCF samples with other loading weights, Figure S5: Frequency dependence of the C_0_ of the SCF samples with a loading weight of 40% (a) as well as the SCF1-1 sample with different loading weights (b), Table S1: Chemical compositions of pristine SiC_nw_ and SCF hybrids.
