# Supplementary material for: Controllable Fabrication of SiC@C-Fe3O4 Hybrids and Their Excellent Electromagnetic Absorption Properties
Source: Nanomaterials (Basel). 2021 Dec 18;11(12):3438. doi: 10.3390/nano11123438 (PMC8706827; doi:10.3390/nano11123438)
Supplement: Supplementary file 1 [file nanomaterials-11-03438-s001.zip › nanomaterials-1472434-supplementary.pdf]

Supplementary Materials

Controllable Fabrication of SiC@C-Fe_3O_4 Hybrids and Their Excellent Electromagnetic Absorption Properties

Liqun Duan [1,*], Xiaoqing Dai [1], Fan Wu [2,*], Aming Xie [2], Jian-An Wu [1], Minqian Sun [1] and Yilu Xia [1]

[1] State Key Laboratory of Disaster Prevention & Mitigation of Explosion & Impact, Army Engineering University of PLA, Nanjing 210007, China; dxq38@sohu.com (X.D.); uniquewujianan@163.com (J.-A.W.); emmassun@hotmail.com (M.S.); xiayilu_lugongda@163.com (Y.X.)

[2] School of Mechanical Engineering, Nanjing University of Science & Technology, Nanjing 210094, China; xieaming@njust.edu.cn

* Correspondence: duanliqun5990379@163.com (L.D.); wufan@njust.edu.cn (F.W.)

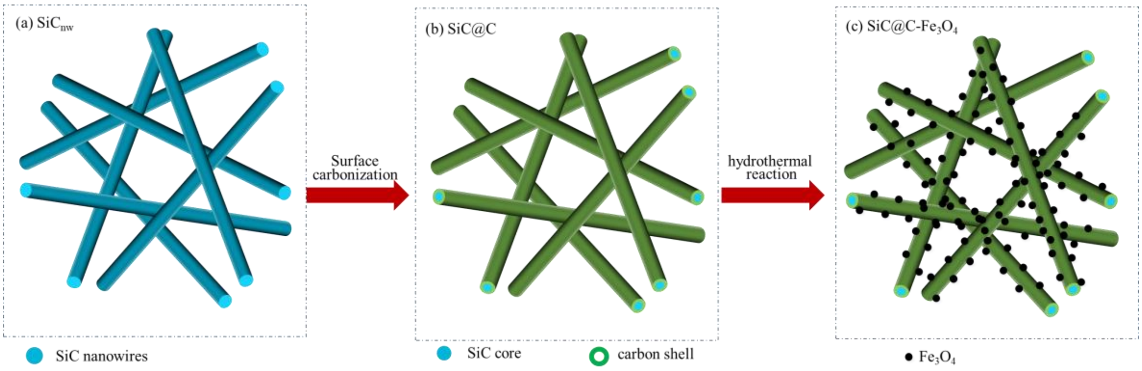

Figure S1. Schematic illustration for fabrication of SCF hybrids.

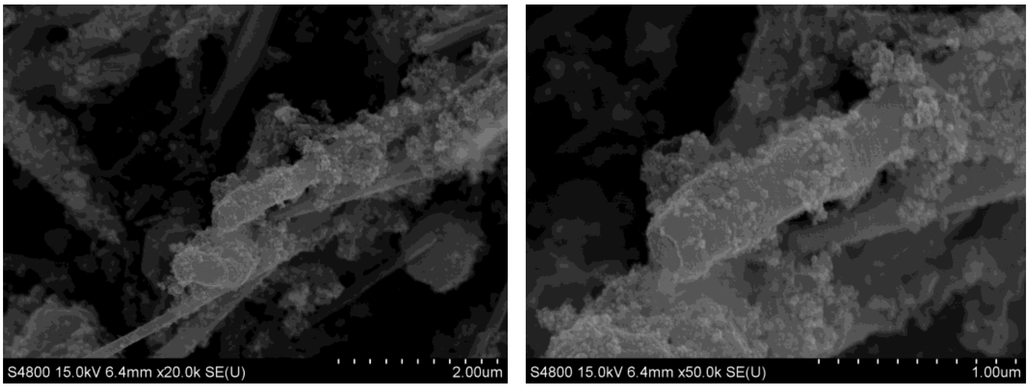

Figure S2. SEM images of SCF1-1 sample

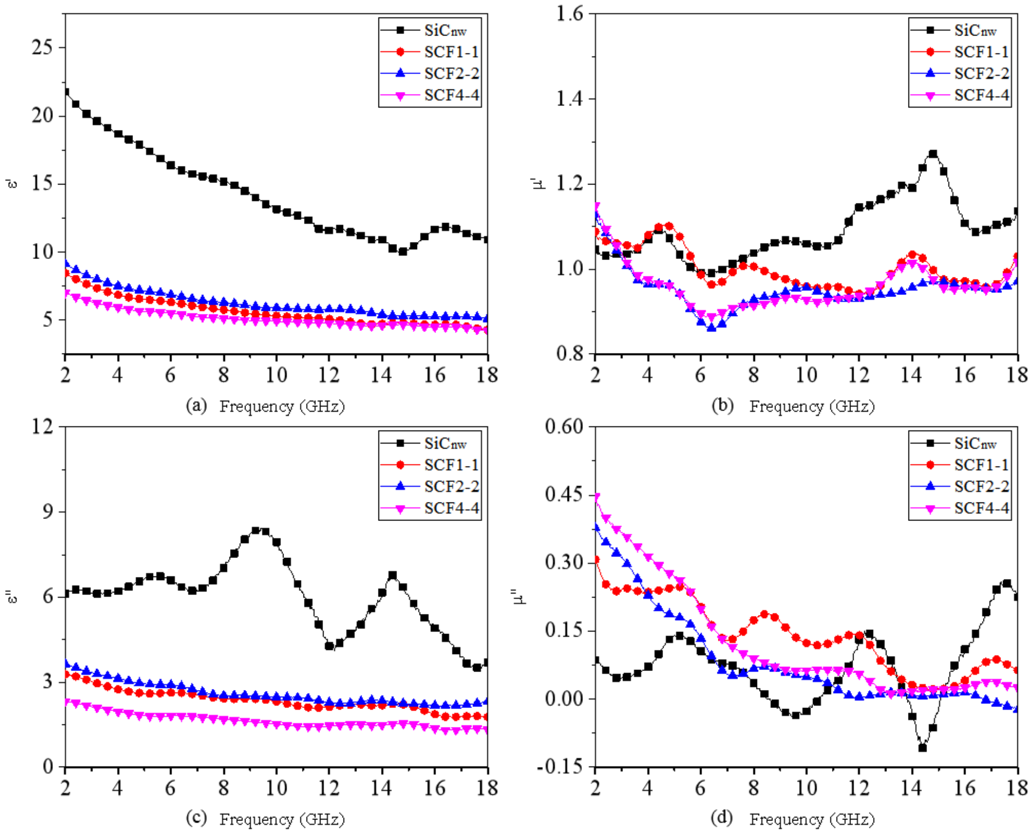

Figure S3. The complex permittivity and permeability characterizations of SiC_{nw} and SCF samples with the same loading weight of 40%.

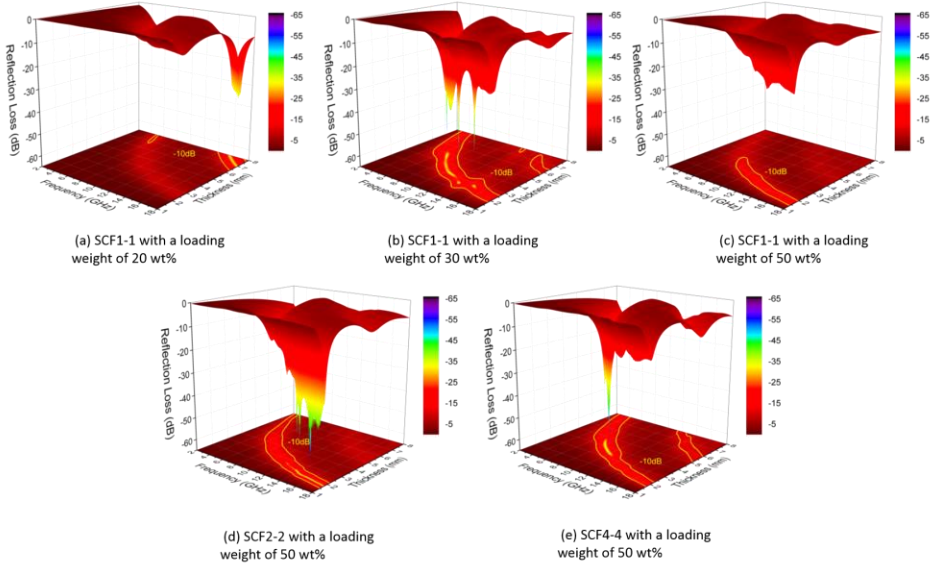

Figure S4. 3D-RL plots of the SCF samples with other loading weights

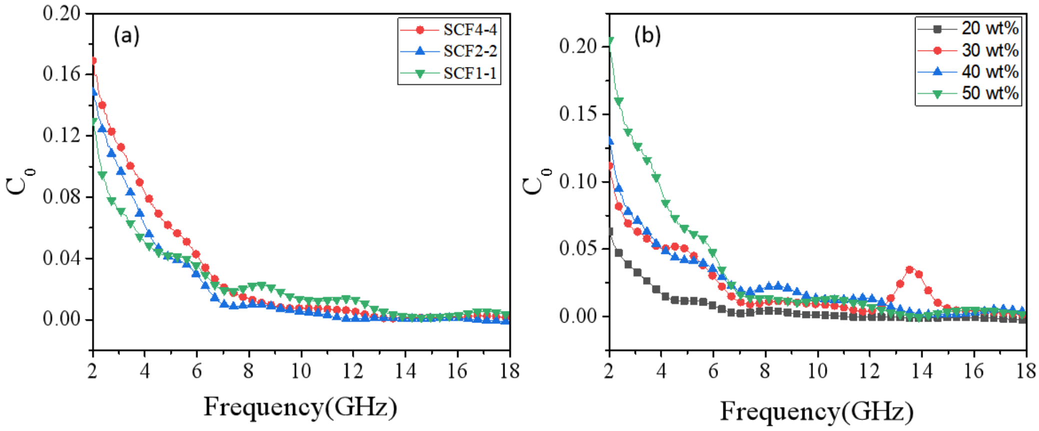

Figure S5. Frequency dependence of the C_0 of the SCF samples with a loading weight of 40% (a) as well as the SCF1-1 sample with different loading weights (b).

Table S1. Chemical compositions of pristine SiC_{nw} and SCF hybrids by EDS technique

Samples	C (at.%)	O (at.%)	Si (at.%)	Fe (at.%)	* (at.%)
SiC_{nw}	44.17±12.00	14.37±2.77	41.42±9.23	-	0.04±0.04
SCF1-1	58.97±6.49	18.46±1.34	15.10±3.39	7.47±3.02	-
SCF2-2	51.83±3.86	23.11±6.85	13.50±6.85	11.56±0.95	-
SCF4-4	31.77±2.59	35.73±1.75	6.65±0.41	25.85±1.52	-

* indicates impurity elements, such as Ca, which is not detected every time.
